# Influence of three different closure techniques on leakage pressures and leakage location following partial cystectomies in normal dogs

**DOI:** 10.1002/vms3.1137

**Published:** 2023-06-08

**Authors:** Jason M. Haas, Daniel J. Duffy, Allison Kendall, Yi‐Jen Chang, George E. Moore

**Affiliations:** ^1^ Department of Clinical Sciences, College of Veterinary Medicine North Carolina State University Raleigh North Carolina; ^2^ Veterinary Administration, College of Veterinary Medicine Purdue University West Lafayette Indiana

**Keywords:** canine, closure techniques, leakage pressures, partial cystectomies

## Abstract

**Background:**

Transitional cell carcinoma (TCC) is the most common neoplasia affecting the canine urinary bladder. Partial cystectomy, when used adjuctively with medical management, has been shown to meaningfully extend medial survival time. Surgical stapling devices have a wide variety of uses and advantages over traditional closure methods and, to date, investigation into their use in canine partial cystectomies has not been documented.

**Objective:**

To determine the influence of three closure techniques on ex vivo leakage pressures and leakage location following canine partial cystectomy.

**Methods:**

Specimens were assigned to one of three closure techniques: simple continuous appositional closure with 3‐0 suture, closure with a 60 mm gastrointestinal stapler with a 3.5 mm cartridge, and placement of a Cushing suture to augment the stapled closure, with each group containing 12 specimens. Mean initial leakage pressure (ILP), maximum leakage pressure (MLP), and leakage location at the time that ILP was recorded were compared between groups.

**Results:**

Oversewn stapled constructs leaked at significantly higher ILP (28.5 mmHg) than those in the sutured (17 mmHg) or stapled (22.8 mmHg) group, respectively. MLP was greater in the oversewn stapled construct group compared to other groups. Leakage was detected in 97% partial cystectomies, with leakage occurring from the needle holes in 100% of the sutured closure group, from the staple holes in 100% of the stapled only group, and from the incisional line in 83% and from bladder wall rupture in 8% of the augmented staple closure group. All closure methods withstood normal physiologic cystic pressures.

**Conclusions:**

Placement of a Cushing suture to augment stapled closures improved the ability of partial cystectomies to sustain higher intravesicular pressures compared with sutured or stapled bladder closures alone. Further in vivo studies are required to determine the clinical significance of these findings and the role of stapling equipment for partial cystectomy, as well as the clinical significance of suture penetration through the urinary bladder mucosa during closure.

## Graphical abstract

Placement of a Cushing suture to augment stapled closures improved the ability of partial cystectomies to sustain higher intravesicular pressures compared with sutured or stapled bladder closures alone in an ex vivo model. These results provide evidence to support placement of a Cushing suture to augment the staple line following stapled partial cystectomy in dogs.

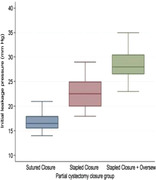

## 1 INTRODUCTION

Neoplastic disease affecting the canine urinary bladder accounts for approximately 2% of all reported neoplasms with transitional cell carcinoma (TCC) identified as the most common form affecting the canine urinary bladder (Knapp et al., 2014; Mutsaers et al., 2003). While the trigonal region of the urinary bladder is the most frequent site of TCC reported in dogs, TCC has also been associated with urethral involvement in 56% and the prostate in 29% of clinically affected dogs (Knapp et al., 2014; Mutsaers et al., 2003;). The apex of the urinary bladder is also a location affected by TCC in dogs, with this location being amenable to surgical removal via partial cystectomy (Norris et al., 1992; Stone et al., 1996).

Although curative intent surgery is often not possible, partial cystectomy has been advocated as an important treatment option for use in targeted multimodal therapy to address nontrigonal TCC. Partial cystectomy is performed to alleviate the clinical signs associated with the apical mass lesion such as dysuria, haematuria and pollakuria with the intent of achieving local control of the tumour and delay onset of metastasis if not already present at the time of surgery (Norris et al., 1992; Stone et al., 1996). Partial cystectomy for the sole management of canine TCC has been shown to extend median survival time (MST) by approximately 365 days when complete excision of a nonmetastatic lesion is achieved and by approximately 120 days if ≥50% of the lesion is excised (Marvel et al., 2017; Norris et al., 1992). Bradbury et al. (2021) found that when partial cystectomy was used adjunctively with concurrent medical management including chemotherapy and or a COX inhibitor, MST was extended to 498 days.

Following open partial cystectomy, the urinary bladder heals quickly with full‐thickness defects returning to inherent native tissue strength at approximately 14–21 days (Bellah, 1989; Daniel & Richard, 1996). Radasch et al. (1990) demonstrated that use of a single layer appositional pattern during closure established a watertight seal that resisted cystic physiologic pressures and had comparable wound strength to two‐layer closure techniques (Lipscomb, 2012; Thieman‐Mankin et al., 2012). Since seeding of neoplastic cells to other intraabdominal tissues can occur following contamination from urinary bladder leakage, it is imperative that bladder wall closure must be able to withstand physiologic intraluminal pressures in awake dogs, especially those encountered during micturition (Marvel et al., 2017; Norris et al., 1992). Mean intraluminal bladder pressures in nonanaesthetised dogs ranged from 10.9 to 12.3 mm Hg; however, the pressures can vary dramatically during detrusor muscle contraction (McCafferty et al. 2009).

Surgical stapling devices have been utilised for a wide variety of applications in veterinary surgery involving the pulmonary, gastrointestinal, vascular, and hepatobiliary systems (Bellah, 1994; Borenstein et al., 2004; Brissot et al., 2003; Clark & Pavletic 1991; Coolman et al., 2000, 1999; Corti et al., 2000; Duffy et al., 2020a, 2021; Larue et al., 1987; Lewis et al., 1990). Benefits associated with use of stapling devices include but are not limited to decreased intraoperative times, preservation of local vasculature, decreased abdominal contamination, creation of an immediate luminal seal, and increased bursting strength compared to hand‐sewn sutured anastomoses (Ballantyne et al., 1985; Tobias 2007; Ullman et al., 1991; White 2008). Comparison of closure techniques in an ex vivo canine typhlectomy model showed that stapled closures resulted in leakage pressures ∼2.5× greater than hand‐sewn closures (Duffy et al., 2020a). One recent study evaluating staple line closure following open canine partial gastrectomy demonstrated that stapled closures augmented with suture reinforcement along the stapled line showed similar maximum leakage pressures compared to closures using a double‐layer suture closure technique (Duffy et al., 2021). This information is of importance to small animal surgeons performing partial cystectomy for apical TCC to allow informed decision making for the use of staple devices and assess their effect on cystic leakage pressures in dogs. Use of a linear stapling device may also have the advantages of en‐bloc resection in an open or laparoscopic setting which may decrease the occurrence of cellular metastasis to the abdominal cavity. To our knowledge, partial canine cystectomy closure using a surgical stapling device has not been described to date.

The objective of this study was to compare the initial leakage pressure (ILP), maximum leakage pressure (MLP), and leakage location between three different closure methods for partial cystectomy closure in an ex vivo model. Our null hypothesis was that there would be no difference in ILP, MLP and leakage location regardless of closure techniques for closure of partial cystectomy sites in dogs.

## 2 MATERIALS AND METHODS

### 2.1 Specimens preparation

Intact canine lower urinary tracts including the bladder, proximal urethra and distal ureters were harvested from 42 healthy adult dogs weighing between 24–34 kg. Dogs were serially obtained following consented donation from a local animal shelter immediately following IV euthanasia of 1 mL/5 Kg sodium pentobarbital (Euthasol; Virbac AH, Fort Worth, TX) for reasons unrelated to this study. An IACUC protocol was not required by our institution for this study due to secondary use of cadaveric tissues. Dogs were excluded from the study if there was any history of cystic disease (cystic calculi, urinary tract infection, cystic neoplasia, bladder trauma) within 2 months of specimen collection or there was visual evidence of urinary tract pathology. Following bladder collection, all residual urine was evacuated from the lumen, and specimens were wrapped in saline (0.9% NaCl) soaked gauze, and individually stored within a thermostatically controlled environment at 4°C based on the results of a prior study in dogs (Duffy et al., 2020b). Prior to testing, all bladders were allowed to reach room temperature (21°C) and were tested within 24 h following euthanasia.

### 2.2 Study randomisation

A total of 42 (*n* = 42) intact urinary bladders were used during the study. Six (*n* = 6) intact bladders were assigned to a control group for assessment of procedural methodology and specimen testing. Thirty‐six canine urinary bladders were randomly assigned to 1 of 3 equally sized experimental groups (*n* = 12/group) using computer software (Research Randomizer; Lancaster, PA. Available at: randomizer.org. Accessed Aug 6, 2020.

### 2.3 Surgical procedure

After careful dissection and removal of periurethral fat and periureteral dissection, any bladder with visual defects or an insufficient trigonal region were excluded. A connecting tubing adapter (PFLLAL, SurgiVet; Smiths Medical ASD, St Paul, MN) was then inserted into the trigone of the urinary bladders and secured using 2 circumferential sutures of 2‐0 polydioxanone (0 PDS; Ethicon, Somerville, NY). A surgeon's knot was placed using a total of 4 throws to secure the adapter in place and suture cut 0.5 cm from the knot. Both ureters were then ligated 1 cm from their respective insertions into the trigone using the same methodology.

For the sutured group, two atraumatic Doyen clamps were placed at the junction of the cranial one‐third and the caudal two‐thirds of the evacuated canine urinary bladder. The urinary bladder was then transected between the adjacent Doyen clamps using straight Metzenbaum scissors. The partial cystectomy site was closed using 3‐0 USP monofilament copolymer of glycolic acid and trimethylene carbonate (Biosyn; Medtronic, Mansfield, MA) on a swaged SH 22 mm ½ circle taper needle in a simple continuous appositional pattern with bites 2–3 mm apart and 2–3 mm from the edge of the incision. Square knots were performed followed by 3 throws at the initiation and termination of the suture line.

For the stapled alone group, a 60 mm linear GIA stapling device (DST Series; Medtronic, Mansfield, MA) preloaded with a blue 3.5 mm staple cartridge (Medtronic, Mansfield, MA) was positioned transversely across the junction of the cranial one‐third and the caudal two‐thirds of the undistended bladder. Once the stapling handpiece was fully engaged and the stapler handpiece seated, manual pressure was applied to close and lock the jaws in place for a precompression time of 10 s. The manual firing knob was then pushed cranially which ejected 2 staggered rows of staples that compressed tissues to 1.5 mm. The integrated knife was used to cut between the two separate rows of staples. Following stapled partial cystectomy, the staple line was evaluated for evidence of staple malformation or failure of the partial cystectomy as would be performed in clinical cases.

For the stapled plus oversew group, partial cystectomies were performed as described for the staple group alone. Following stapled partial cystectomy closure using the stapling hand piece, the staple line was then oversewn using monofilament 3‐0 Biosyn (Biosyn; Medtronic, Mansfield, MA) on a swaged SH 22 mm ½ circle taper needle placed using a Cushing (inverting) suture pattern placed 2 mm distal to the staple line. All sutures were placed 2–3 mm apart and a square knot followed by 3 throws was used to start and finish the suture line in the oversew. If required during suturing, inversion of the staple line was performed by the use of mosquito haemostats.

For control specimens, no partial cystectomy was performed. Control specimens were used to evaluate intraluminal pressures resulting in catastrophic bladder failure for assessment of intraluminal pressure testing methods. A single board‐certified small animal surgeon (D.J.D.) familiar with and trained on the use of stapling devices performed all partial cystectomies and resultant closures regardless of randomised group assignment and was assisted by a trained surgical assistant. Tissues were kept moist during testing with room temperature (21°C) 0.9% NaCl administered from a spray bottle.

### 2.4 Evaluation of bladder leakage and failure location

Partial cystectomy site closures were tested after being connected to a 3‐way stopcock (Disconfix; Braun Medical, Bethlehem, PA) adjoined to a tubing adaptor that had been previously placed in the urethral trigone (Duffy et al., 2019). The remaining ports were connected to a fluid line (Hospira, Elk Grove Village, IL) that was used to infuse the urinary bladder while the other was connected to a pressure transducer (Deltran II; Utah Medical Products, Midvale, UT) to allow assessment of intraluminal pressures. A 5 L bag of electrolyte solution (Vetivex; Dechra Veterinary Products, Overland Park, KS) to which 10 mL of coloured blue dye (Methylene blue; Kordon LLC, Hayward, CA) had been added was connected to a fluid pump (HESKA VET/IV 2.2; Heska, Loveland, CO). The pressure transducer was serially connected to a Passport 12 pressure monitor (Mindray North America, Mahwah, NJ) and zeroed/calibrated at the same level as the bladder at the start of each test. Fluid was infused at a rate of 999 mL/h based on the methods of prior studies (Chu et al., 2020; Duffy et al., 2018, 2019; Hansen & Monnet 2012; Spiller et al., 2015). During fluid infusion, the cystectomy site and urinary bladder surface were monitored for leakage by a single study investigator. The ILP and MLP were recorded in mm Hg. ILP was defined as the intraluminal pressure at which the coloured saline solution was first observed to leak extraluminally. MLP was defined as the intraluminal pressure that caused catastrophic failure where there was an acute drop of >30% mm Hg or there was plateau of the intraluminal pressure readings for >8 s in duration. The location of leakage was defined as the location at which dyed fluid was observed to leak extraluminally at the level of the suture holes, staple holes, along the incisional line, or due to rupture of the bladder wall.

### 2.5 Statistical analysis

A pilot study was performed to determine the methods for canine bladder collection, preparation and dissection, partial cystectomy regardless of group, suturing, and determination of ILP, MLP and leakage location which was then refined for use in the current study. A prospective power analysis determined that a sample size of ≥10 specimens per group was required to detect a difference of ≥20 ± 5 mm Hg leakage pressure between groups, using a power of 80%, a confidence interval of 95% and an alpha‐error rate of 5%. Data was assessed for normality using the Shapiro–Wilk test. Comparison of ILP and MLP within groups was performed using Scheffe's adjustment. Specimen weight (g) was compared using analysis of variance. Summary statistics for ILP (mm Hg) and MLP (mm Hg) were reported as median (range). A Fisher's exact test was used to assess differences in leakage location by group, with a *p* value of ≤0.05 considered statistically significant. Computed analysis was performed using Stata/SE v.15.0 (StataCorp; College Station, TX).

## 3 RESULTS

All partial cystectomies were successfully performed and leakage pressure testing was accomplished without observed technical error during specimen harvest, partial cystectomy and assigned closure method. No specimens were rejected at the time of collection with all bladders included in the final statistical model. Mean canine bladder weight was 23.88 ± 4.36 g with no difference between groups (*p* = 0.819).

### 3.1 Leakage pressure testing

**FIGURE 1:**
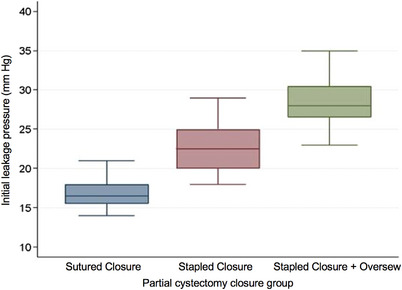
Box and whisker plot illustrating ILP for different experimental groups following partial cystectomy closure. There was a significant difference in ILP among groups (*p* < 0.0001), with the augmented stapled closures leaking at higher ILP than the suture and staple alone groups (*p* < 0.001), respectively. The staple alone group leaked at higher ILP than the suture group (*p* < 0.001). Boxes represent the interquartile (25th to 75th percentile) range, the horizontal line in each box represents the median, and whiskers represent the maximum and minimum values. Abbreviations: ILP, initial leak pressure.

**FIGURE 2:**
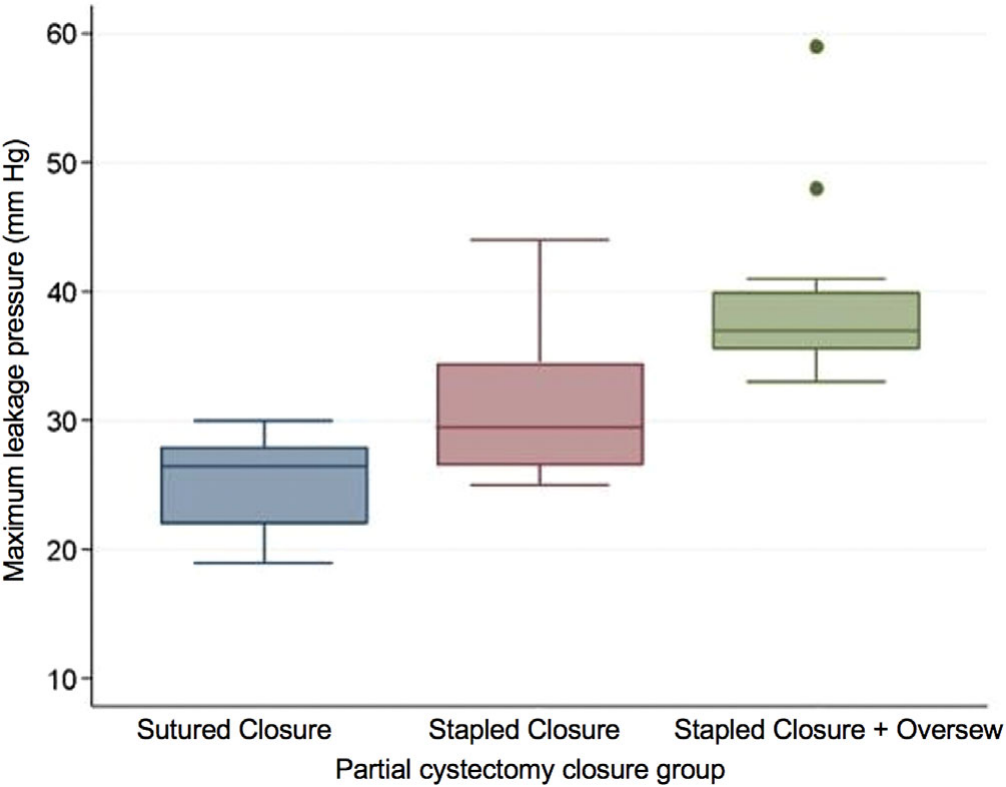
Box and whisker plot illustrating MLP for experimental groups. Stapled closures augmented with a sutured oversew had significantly greater MLP compared to sutured (*p* < 0.0001) or stapled (*p* < 0.005) partial cystectomy closure alone, respectively. Boxes represent the interquartile (25th to 75th percentile) range, the horizontal line in each box represents the median, whiskers represent the maximum and minimum values, and the circles represent an outlier within the data set. Abbreviations: MLP, maximum leakage pressure.

There was a difference in ILP among groups (*p* < 0.0001). Stapled partial cystectomy augmented with an inverting Cushing suture pattern leaked at higher ILP when compared to sutured (*p* < 0.0001) or stapled closures (*p* < 0.0001) alone, respectively (Figure 1). ILP was greater for stapled alone compared to sutured partial cystectomy closures (*p* < 0.001). The ILP of control specimens were greater compared to all experimental groups (*p* < 0.0001). ILP can be seen in Table 1. MLP differed among experimental groups (*p* < 0.0001). Stapled closures augmented with a sutured oversew had significantly greater MLP compared to sutured (*p* < 0.0001) or stapled (*p* < 0.005) partial cystectomy closure alone, respectively. There was no difference in MLP between sutured versus stapled closures alone (*p* = 0.059; Figure 2). MLP of control specimens was greater compared to all other groups (*p* < 0.0001). MLP can be seen in Table 1.

**TABLE 1:** Median (range) values for ILP and MLP of partial cystectomies closed with three different closure techniques in dogs.

Pressure (mm Hg)	Sutured closure	Stapled closure	Stapled closure + oversew
ILP	17 (14.91–19.09)	22.75 (19.34–26.16)	28.5 (24.82–32.18)
MLP	25.25 (21.76–28.74)	31.08 (25.42–36.74)	39.33 (31.94–46.72)

Abbreviations: ILP, initial leak pressure; MLP, maximum leakage pressure.

### 3.2 Leakage location

Leakage location differed among groups (*p* < 0.001) and all leakage locations were recorded at the time that ILP was recorded. Partial cystectomies leaked from the incisional line in 10 of 36 (28%) constructs, from a suture hole in 13 of 36 (36%) constructs and from a staple hole in 12 of 36 (33%) constructs. There was no observed leakage occurring in one (3%) of the experimental constructs with failure seen in this construct due to a full‐thickness bladder wall rupture immediately adjacent to the trigone. Leakage occurred from a suture hole in 12 of 12 (100%) constructs in the sutured closure group and at one or more staple holes in 12 of 12 (100%) constructs in the stapled only group. Leakage occurred from the incisional line in 10 of 12 (83%) and by full‐thickness bladder wall rupture in 1 of 12 (8%) of constructs in the stapled partial cystectomy closure augmented with an inverting Cushing suture. Six of six (100%) specimens in the control group failed by bladder wall rupture.

## 4 DISCUSSION

In this study, we evaluated the effect of three different closure techniques following partial cystectomy in a canine ex vivo model. We failed to reject our null hypothesis, as there was a significant difference in ILP, MLP and leakage location based upon closure method of experimental partial cystectomy. Stapled closures augmented with a suture oversew in a Cushing pattern had an ILP that were 1.7‐fold and 1.3‐fold greater than sutured or stapled closures alone, respectively. Suture augmented stapled partial cystectomy had MLP that were 1.5‐fold and 1.2‐fold greater than sutured or stapled closures alone, respectively. Leakage location differed between groups with leakage exclusively at the suture holes in the sutured closure group, exclusively at the staple holes in the stapled closure group, and primarily at the incisional line in stapled closures augmented with a suture oversew.

A single layer appositional closure of the canine urinary bladder is effective at creating a watertight seal (Brissot et al., 2003; Lipscomb, 2012; McCafferty et al., 2009). Due to the potential for abdominal seeding from free‐floating TCC cells in the urine or tissues, there has been an emphasis on the need for minimal to no leakage at the time of surgery in the peri‐ or postoperative period in dogs (Lipscomb 2012; Marvel et al., 2017; Norris et al., 1992; Radasch et al., 1990; Thieman‐Mankin et al., 2012). In our study, there was a 1.3‐fold increase in ILP when a stapling device was used compared to sutured closures alone. Furthermore, stapled closures augmented with a suture oversew using a Cushing pattern had significantly higher ILP when compared to sutured or stapled closure methods alone, with a 1.7‐fold and 1.3‐fold increase, respectively. These findings are in agreement with previous studies evaluating various closure techniques utilising staple line reinforcement in both human and dogs (Duffy et al., 2020a, 2021; Karakoyun et al., 2015). Postulated reasons for these observed findings may be due to the use of a GIA stapler leading to an inverting closure that is then augmented with an oversew using a Cushing pattern which then further establishes serosal‐to‐serosal contact that further strengthens the watertight seal (Ellison et al., 2019; Hany & Ibrahim 2018; Ser et al., 2010).

Stapled closures augmented with a suture oversew using a Cushing pattern had significantly higher MLP when compared to sutured or stapled closures alone with a 1.5‐fold and 1.2‐fold increase in MLP, respectively. In our experiment there was no difference in MLP between sutured or stapled closures alone. Since the urinary bladder is a variable pressure system, with relatively low pressures during the bladder filling and greater pressures encountered during active micturition and detrusor muscle contraction, increases in MLP may further decrease the risk of partial cystectomy site dehiscence and resultant leakage. Although postoperative decompression may include placement of an indwelling urinary catheter to minimise intraluminal pressures during the initial phases of wound healing, dislodgement of the catheter or concern for urothelial irritation at the partial cystectomy site make MLP values of interest clinically. Suture augmentation following stapled partial cystectomy closures should be considered to ensure maximal resistance to leakage of urine and decrease the occurrence of seeding TCC cells to the peritoneal cavity. The effect of laparoscopic partial cystectomy closure using a stapling device on minimising abdominal contamination and its effect on en‐bloc resection in attempts to decrease intraabdominal metastasis remains unknown and is an area for future investigation.

Although intraluminal pressure within the normal canine urinary bladder are typically low, ranging from 0 to 12.3 mm Hg, differences among breeds, bladder pathology, rate of luminal filling, hydration status, and renal filtration pressures may contribute to a wide range of intraluminal pressures being encountered during active micturition and urination (Conzemius et al., 1995; Fetner & Prittie, 2012; Hill, 2015; McCafferty et al., 2009). In order to allow for storage of urine within the intact urinary bladder between micturition events, the urinary epithelium and connective tissues within the bladder wall stretch with smooth muscle restructuring and resultant urine accommodation (Duffy et al., 2018). These physiologic variables allow for low intraluminal pressures to prevent vesicoureteral backflow of urine during storage (Hill, 2015). In our study, none of the experimental constructs leaked <14 mm Hg, signifying that all closure methods may be sufficient in resisting physiologic pressures encountered during luminal filling in normal healthy dogs.

Stapled closure of partial cystectomy sites may be advantageous in the treatment of dogs with apical bladder TCC because the increase in ILP decreases the risk of urine leakage leading to TCC seeding throughout the abdomen. Use of a GIA stapling device deploys two staggered rows of staples on either side of the partial cystectomy site, which may decrease the risk of urine leakage not only from the remaining urinary bladder but also from the resected tissue edge. Stapling devices have been used with increased frequency in canine intestinal surgery with advantages including but not limited to; shorter surgical times, consistent staple placement, decreased iatrogenic tissue trauma, preservation of local blood supply, and ease and repeatability of use with novice surgeons (Ellison et al., 2019; Jardel et al., 2011; Kieves et al., 2018; Tobias, 2007; Ullman et al., 1991). Based on our study, augmentation of the staple line with suture following partial cystectomy may further increase resistance of the urinary bladder closure to extraluminal leakage. Although subjectively assessed, addition of the suture oversew was associated with additional procedural time that would likely be clinically irrelevant in vivo. We recognise that oversewing of the staple line may decrease the functional bladder volume, reservoir capacity of the bladder, and that secondary effects such as pollakiuria, stranguria or haematuria due to internalisation of the partial cystectomy site into the bladder lumen are possible and require further study in dogs.

In our study, leakage location differed among partial cystectomy closure techniques. Partial cystectomy sites closed with suture leaked exclusively from suture holes at sites of needle penetration which is in agreement with the results of prior investigators (Duffy et al., 2018, 2019, 2020a; Kieves & Krebs, 2017; Kieves et al., 2018; Montel et al., 2017). The relevance of these findings and resultant leakage location remains unclear in ex vivo models since the effect of an early fibrin seal development and early mucosal regeneration are not able to be assessed. Leakage location in the stapled only group was observed in partial cystectomy closures following deployment of a 3.5 mm stapling cartridge with leakage circumferentially around the staple holes. Our results are similar to those of prior leakage pressure assessments in comparative gastrointestinal studies in dogs and people (Chu et al., 2020; Duffy et al., 2020a, 2021; Karakoyun et al., 2015). Closure of canine partial cystectomy sites using a stapling device followed by suture augmentation demonstrated leakage primarily along the length of the incisional line rather than at sites of needle or staple penetration, which mirrors prior studies investigating closure techniques in luminal tissues in dogs (Jardel et al., 2011; Sumner et al., 2019). This finding is likely due to the inverting nature of the initial staple line followed by augmentation using suture involuting the cut edge of the partial cystectomy into the bladder lumen. We recognised in vivo that bleeding from the internalised staple line into the bladder lumen may occur but this was unable to be assessed. It should be noted that the GIA DST stapling device deploys staples in an inverting configuration, which further increases serosal‐to‐serosal contact and therefore strengthens the watertight seal (Ellison et al., 2019). In a setting of apical bladder pathology, it is plausible that mucosal inversion into the bladder lumen may be important since neoplastic cells can be found histologically throughout the entire bladder mucosa. Exposure of neoplastic tissue may predispose to abdominal adhesion formation and to possible spread of TCC cells to abdominal viscera. These speculative hypotheses, however, require further evaluation in vivo.

This study is inherently limited in its application to in vivo studies since evaluation of normal physiologic processes such as fibrin seal development and its contribution to initial wound strength, inflammation, coagulation, and stages of wound healing cannot be assessed using cadaveric models. It is important to note that we utilised canine urinary bladders free of gross disease or pathology. Alteration of urinary bladder wall architecture by neoplastic involvement may decrease the inherent strength of cystic tissues at the incisional line leading to leakage at lower pressures compared to those observed in our model. Avoidance of urine leakage into the peritoneum is crucial for decreasing the prevalence of TCC translocation, thus, further studies evaluating leakage pressures in either grossly or microscopically affected tissue and live tissue is required prior to consideration and safe application of these stapling methods in vivo. Unlike sutured partial cystectomy techniques using absorbable suture materials, staples used in this study were nonabsorbable and implanted into the bladder wall. We recognise that use of these devices may serve as a reservoir for bacterial adherence, biofilm development and predisposition to cystic calculi formation or urinary tract infection, potentially contributing to increased morbidity postoperatively in vivo. These implants may also limit the ability to use staging modalities such as computed tomographic evaluation by causing imaging artefacts such as beam hardening and scatter, motion, and edge effects. The rate of luminal filling during leak pressure testing was much faster than that encountered during normal physiologic filling of the urinary bladder in dogs. In the normal canine urinary bladder, the urinary epithelium and connective tissues within the bladder wall stretch and smooth muscle restructures leading to urine accommodation within the bladder lumen, this could not be assessed during our study. An additional limitation in this study is that partial cystectomy closures with suture alone leaked solely at the needle holes which was likely due to the needle penetrating the urinary bladder mucosa during suture placement. The authors recognise that in live patients, care is taken to not penetrate the mucosa during closure to avoid this complication. Although stapled partial cystectomy closures also involved penetrating the urinary bladder mucosa, ILP was shown to be significantly higher in stapled closures versus sutured closures. Further studies are needed to compare partial cystectomy closure with sutures that do not penetrate the urinary bladder mucosa versus stapled closures in order to understand whether the strength of stapled closures might offset the concern that the urinary bladder mucosa is penetrated.

In conclusion, the results of this in vitro study evaluating canine partial cystectomy closure using a 3.5 mm GIA DST stapling device augmented within a Cushing suture increased ILP and MLP compared to sutured or stapled closures alone and may represent a possible closure method following partial cystectomy in dogs. A Cushing suture pattern to augment stapled closure improved the ability of partial cystectomies to sustain intravesicular pressures and increased ILP by 1.7‐fold and 1.3‐fold and MLP by 1.5‐fold and 1.3‐fold respectively compared to use of sutured or stapled closure techniques alone. These results provide evidence to support placement of a Cushing suture pattern to augment the staple line following stapled partial cystectomy in dogs. In vivo studies are required to determine the clinical significance of these findings and the role of stapling equipment for laparoscopic partial cystectomy in dogs.

## AUTHOR CONTRIBUTIONS

Jason Haas: Visualisation (equal); writing – original draft preparation (lead); writing – review & editing (equal). Daniel Duffy: Conceptualisation (lead); data curation (equal); funding acquisition (lead); investigation (equal); methodology (lead); project administration (lead); resources (lead); supervision (lead); validation (lead); visualisation (equal); writing – original draft preparation (supporting); writing – review & editing (equal). Allison Kendall: Conceptualisation (supporting). Yi‐Jen Chang: Data curation (equal); investigation (equal); methodology (supporting). George Moore: Formal analysis (lead).

## FUNDING

The authors declare that there are no financial relationships that may affect the results of this study.

## CONFLICT OF INTEREST STATEMENT

The authors declare that they have no conflicts of interest.

## ETHICS STATEMENT

The authors confirm that the ethical policies of the journal, as noted on the journal's author guidelines page, have been adhered to. No ethical approval was required as this study was performed with the secondary use of cadaveric tissues.

### PEER REVIEW

The peer review history for this article is available at https://publons.com/publon/10.1002/vms3.1137.

## Data Availability

The data that support the findings of this study are available from the corresponding author upon reasonable request.
